# E3 Ubiquitin Ligases: Potential Therapeutic Targets for Skeletal Pathology and Degeneration

**DOI:** 10.1155/2022/6948367

**Published:** 2022-09-27

**Authors:** Ruiyin Zeng, Yuan Xiong, Ze Lin, Adriana C. Panayi, Yun Sun, Faqi Cao, Guohui Liu

**Affiliations:** ^1^Department of Orthopaedics, Union Hospital, Tongji Medical College, Huazhong University of Science and Technology, Wuhan 430022, China; ^2^Hubei Province Key Laboratory of Oral and Maxillofacial Development and Regeneration, Wuhan 430022, China; ^3^Division of Plastic Surgery, Brigham and Women's Hospital and Harvard Medical School, Boston, MA, USA; ^4^Department of Neurosurgery, Union Hospital, Tongji Medical College, Huazhong University of Science and Technology, Wuhan, China

## Abstract

The ubiquitination-proteasome system (UPS) is crucial in regulating a variety of cellular processes including proliferation, differentiation, and survival. Ubiquitin protein ligase E3 is the most critical molecule in the UPS system. Dysregulation of the UPS system is associated with many conditions. Over the past few decades, there have been an increasing number of studies focusing on the UPS system and how it affects bone metabolism. Multiple E3 ubiquitin ligases have been found to mediate osteogenesis or osteolysis through a variety of pathways. In this review, we describe the mechanisms of UPS, especially E3 ubiquitin ligases on bone metabolism. To date, many E3 ubiquitin ligases have been found to regulate osteogenesis or osteoclast differentiation. We review the classification of these E3 enzymes and the mechanisms that influence upstream and downstream molecules and transduction pathways. Finally, this paper reviews the discovery of the relevant UPS inhibitors, drug molecules, and noncoding RNAs so far and prospects the future research and treatment.

## 1. Introduction

The ubiquitin-proteasome system (UPS) is comprised of several key components: ubiquitin (Ub), Ub-activating enzyme (E1), Ub-conjugating enzyme (E2), ubiquitin ligase (E3), deubiquitinating enzyme (DUB), and proteasome. UPS is an enzymatic cascade reaction that mediates the labeling of target proteins with ubiquitin tags, leading to their degradation via the proteasome pathway. The entire ubiquitination process can be briefly described as follows: Step 1: E1 activates ubiquitin and forms an E1-ubiquitin intermediate. This process requires the consumption of ATP. Step 2: ubiquitin is transferred from E1s to E2s, forming an E2-ubiquitin intermediate. Step 3: the E3s first recognize the target protein to be degraded and then recognize the E2-ubiquitin intermediate, forming a complex containing the E2-ubiquitin intermediate, the E3s, and the target protein, and finally transfer the activated ubiquitin from E2s to the target protein. Step 4: the E2 enzyme and E3 enzyme are released from the complex, leaving the ubiquitin-tagged target protein. Step 5: the above process is repeated until multiple ubiquitin molecules are attached to the target protein to form a ubiquitin chain. Step 6: the ubiquitinated target protein is recognized and degraded into small fragments by the 26S proteasome. This process can be reversed by a group of proteases called the deubiquitinating enzymes (DUBs) which hydrolyze the peptide bond that links the target protein and ubiquitin [1].

During bone formation and reconstruction, osteogenic and osteoclastic activities need to be precisely coordinated in order to maintain bone homeostasis. This is mainly mediated through three cell lineages: osteoblasts, osteoclasts, and osteocytes [2]. Osteoclasts differentiate from macrophages and monocytes in the human hematopoietic system and play essential roles in bone resorption [3]. Osteoblasts differentiate from mesenchymal stem cells (MSC) and synthesize, secrete, and mineralize bone matrix. Osteoblasts are the main functional cells in bone formation [4]. Osteocytes are the most common cells in mature bone tissue and are isolated from osteoblasts, which sense and transmit signals and secrete cytokines. These cells constitute the basic multicellular unit (BMU) that performs the bone reconstruction cycle [5]. Thus, the differentiation, function, and interaction of these cells are critical for regulating bone remodeling and maintaining bone homeostasis. E3 ubiquitin ligases have been found to influence osteoblasts and osteoclasts from a variety of mechanisms [6]. Therefore, regulating the relevant E3 ubiquitin ligases is an ideal approach for the treatment of the skeletal disorder.

In this review, we briefly describe the structure and function of the UPS, the mechanism of action of E3 ubiquitin ligases in bone metabolism regulation, and the E3 ubiquitin protein ligase inhibitors currently in use and molecules that are promising targets for future drug therapy.

## 2. Effect of E3 Ubiquitin Ligases in Skeletal Cell Fate and Pathology

E3 ubiquitin ligases can be classified into three major types based on their structures: the “really interesting new gene” (RING) family, the “homologous to E6-AP carboxyl terminus” (HECT) family, and the RING-between-RING-RING (RBR) family [7]. Different ligase domains can have specific ubiquitin transfer modes. For example, the RING E3s act as a scaffold that binds the E2 enzyme and substrate together, and ubiquitin is transferred directly from the E2s to the substrate without forming the E3-ubiquitin intermediate. However, in HECT E3s, an E3-ubiquitin intermediate is formed before ubiquitin is transferred to its substrate. More than 600 types of E3 ligases have been identified in the human genome, which contribute to the specificity of the UPS system [8].

Differentiation of the osteoblast lineage is regulated by a complex signaling pathway. Early osteoblast differentiation is mainly regulated by the BMP-SMAD-RUNX2 pathway. RUNX2 and its downstream molecule Osterix are the paramount osteoblast-specific transcription factors. This pathway triggers the expression of osteoblast phenotype genes and synthesizes bone matrix at a later stage [9]. In addition to this, Hedgehog, JNK, TGF-*β*, and classical Wnt/*β*-catenin signaling pathways are associated with the development of osteoblasts [10]. Osteoblasts then embed in the bone matrix as osteocytes or die at the end of their fate [11]. Many E3 enzymes can regulate these pathways and in turn affect osteogenesis. For example, SMURF1 acts on multiple components of the BMP-SMAD-RUNX2 and MEKK2-JNK-JUNB pathways and inhibits osteogenesis. Cdh1 regulates the MEKK2 pathway to inhibit osteogenesis. SMURF2 downregulates the TGF-*β* pathway, thereby hindering the PI3-kinase-AKT pathway activation, which in turn inhibits osteogenesis. WWP1 inhibits osteogenesis by promoting the degradation of SMAD4, RUNX2, and JUNB ubiquitination in osteoblasts. ITCH negatively regulates osteogenesis through JunB degradation. On the other hand, there are a number of E3 ubiquitinases that could promote osteogenesis. For example, TRIM16 reduces CHIP, therefore alleviates CHIP-mediated degradation of RUNX2, and then enhanced osteogenic. Besides, there are also proteins such as Cbl-b and c-Cbl that positively or negatively regulate bone formation by ubiquitinating the RTK-PI3K-AKT axis and other c-Cbl target proteins. In addition, insulin, through insulin-like growth factor-I (IGF-I), also affects the generation and differentiation of osteoblasts, while Cbl-b inhibits IGF-I-regulated osteogenic differentiation [12] (Table 1).

Osteoclasts are large multinucleated cells derived from the hematopoietic spectrum and regulated by several factors. Among them, the production of M-SCF and RANKL by bone marrow stromal cells and osteoblasts is essential in promoting osteoclastogenesis. M-CSF promotes the proliferation of osteoclast precursors, while RANKL stimulates the differentiation of osteoclast precursors to mature osteoclasts. In addition, the NF-*κ*B and Wnt/*β*-catenin pathways also play an important role during osteoclast differentiation, which is regulated by E3 ubiquitin ligases [13]. For example, SMURF2 promotes osteoclastic differentiation by regulating RANKL expression; TRIM38 and CHIP negatively regulate NF-*κ*B and inhibit osteoclastic differentiation; RNF146 regulates the 3BP2/SRC pathway and Wnt/*β*-catenin pathway and inhibits osteoclastic differentiation; LNX2 promotes activation of the NF-*κ*B and JNK pathways and downregulation of North pathway which enhances osteoclast differentiation (Table 1).

Following, we reviewed the detailed effects of a series of E3 ubiquitin ligases which have been found to regulate the differentiation of osteoblasts and osteoclasts.

### 2.1. SMURF1

SMURF1, which belongs to the Hect family of E3 ubiquitin ligases, interacts with BMP pathway-specific receptor-regulated SMADs to trigger their ubiquitination and degradation, thereby inactivating them. SMADs have three subgroups: receptor-activated SMADs (for example, SMAD1, -2, -3, -5, and -8), common SMADs (for example, SMAD4), and inhibitory SMADs (for example, SMAD6 and SMAD7) [14]. SMURF1 selectively interacts with BMP pathway-targeted SMAD1 and SMAD5 to induce their degradation, thus blocking BMP-SMAD-RUNX2 signal transduction [15]. In addition, SMURF1 and SMAD (SMAD6 or 7) inhibitors synergistically negatively regulate BMP by downregulating activated BMP receptors as well as receptors of R-SMADs [16]. A regulatory circuit exists between RUNX2 and the E3 ligase SMURF1. SMURF1 acts on the C-terminal PY motif of RUNX2 and mediates RUNX2 ubiquitination, while SMAD6 enhances SMURF1-induced RUNX2 degradation [17] and RUNX2 activates SMURF1 transcription in osteoblasts [18].

TGF-*β*1 plays a multifaceted role in regulating osteoblast differentiation. In the early differentiation of osteoblast cells, TGF-*β*1 promotes proliferation and differentiation through the SMAD2/3 pathways [19]. However, TGF-*β*1 promotes the ubiquitination and degradation of TGF-*β*1 type I receptor by inducing SMURF1 and SMURF2, which in turn inhibits osteoblast mineralization during the late stages of osteoblast differentiation [20–22]. Moreover, TGF-*β*1 degrades the C/EBP*β* protein by inducing SMURF1 expression at the transcriptional level, thereby reducing C/EBP*β*-DKK1 and inhibiting matrix mineralization during osteoblast differentiation [23].

RAS-MAPK-ERK signaling pathway also plays a dual role in bone metabolism. Crosstalk exists between the TGF-*β*/BMP-SMAD and RAS-MAPK signaling pathways [24]. TGF-*β* can upregulate the expression of SMURF1 by activating the MAPK-ERK pathway, then increase the proteasome degradation of RUNX2 and SMAD1, and inhibit osteogenic differentiation [25]. Furthermore, SMURF1 can directly interact with MEKK2 and affect the activation of the downstream JNK signal cascade [26].

Tumor necrosis factor (TNF) is a proinflammatory cytokine which is one of the main factors involved in pathological bone loss [27]. One of the mechanisms of TNF in inflammatory bone disease is the induction of the expression of the ubiquitin ligases SMURF1 and SMURF2, thus promoting the ubiquitination degradation of SMAD1/5 and RUNX2 and leading to systemic bone loss [28, 29]. The possible molecular mechanism underlying is that the presence of AP-1, RUNX2, and TNF-*α* activates JNK and ERK, which induces JNK binding of RUNX2 and c-Jun to the SMURF1 promoter, thus promoting SMURF1 transcription [30].

**Figure 1:**
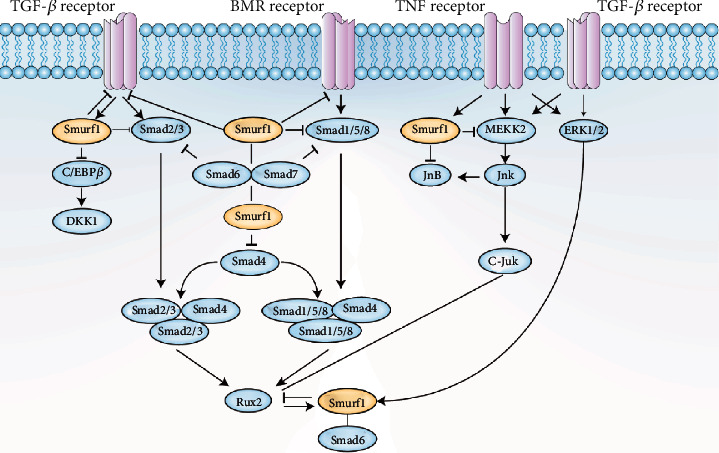
The E3 ubiquitin ligase SMURF1 mediates the ubiquitination and degradation of key factors from BMP/TGF-*β* pathway, NF-*κ*B pathway, MAPK pathway, and other pathways, thereby regulating osteogenic differentiation. Notably, these pathways interconnected with each other and formed a complex regulatory network.

Furthermore, SMURF1 can regulate cell polarity and process formation by targeting the RhoA ubiquitination degradation [31] and negatively regulating MSC proliferation and differentiation by promoting JunB degradation [32]. Continuous PTH treatment can increase SMURF1 expression in osteoblasts, leading to RUNX2 degradation and reducing antiapoptotic signaling in osteoblasts [33] (Figure 1).

### 2.2. SMURF2

SMURF2 is an E3 ligase of the Hect family which mainly regulates TGF-*β*/BMP signaling through a pathway similar to but independent of SMURF1. SMURF2 preferentially targets SMAD1 for ubiquitination and degradation and has weaker affinity for SMAD2 and SMAD3 [34]. In addition, when SMURF2 was coexpressed with R-SMAD and SMAD2, SMURF2 showed the ability to downregulate SMAD4 similarly to SMURF1 [35]. Under IFN*γ* induction, SMURF2 and inhibitory SMADs (such as SMAD7) form a SMAD7-SMURF2 complex, which targets TGF-*β* receptors for degradation and thus bone metabolism [36]. A study showed that SMURF2 mice showed severe osteoporosis with an increased number of osteoclasts. A possible mechanism is that SMURF2-mediated SMAD3 ubiquitination affects the interaction between SMAD3 and vitamin D receptors, which regulates RANKL expression [37]. AKT is one of the key cytokines in bone anabolic signaling [12], and the PI3-kinase-AKT pathway intersects with the BMP pathway. Experiments have shown that AKT enhances RUNX2 expression by inducing SMURF2 ubiquitination and degradation which enhances the stability of the RUNX2 protein [38]. SMURF2 also stimulates chondrocyte maturation during endochondral ossification. Specifically, SMURF2 induces GSK-3 *β* ubiquitination and proteasome degradation, leading to the upregulation of *β*-catenin which promotes endochondral ossification via the Wnt signaling pathway [39].

### 2.3. APC/C^CDC20^ and APC/C^Cdh1^

The anaphase-promoting complex or cyclosome (APC/C) is a multisubunit ubiquitin ligase that regulates multiple cell cycle transitions. Two APC/C activators, Cdc20 and Cdh1, directly bind to APC/C, activate its ubiquitin ligase activity, and contribute to its substrate recognition and specificity [40]. APC/C also has cell cycle-independent functions. APC/C^CDC20^ promoted the osteogenic differentiation of BMSCs by ubiquitination and degradation of p65 [41]. Conversely, the interaction between Cdh1 and SMURF1 enhances Smurf1-mediated ubiquitination of its downstream targets and inhibits osteoblast differentiation by regulating the activity of the MEKK2 pathway [42].

### 2.4. TRAF4

TNF receptor-associated factor 4 (TRAF4), a member of the TRAF family and a ubiquitin ligase in the RING family, plays an important role in the embryogenesis and development of the skeletal system. It was demonstrated that TRAF4 acts as an E3 ubiquitin ligase that positively regulates the osteogenesis of MSCs by mediating the ubiquitination of the K48 linkage of SMURF2 at the K119 locus and leading to its degradation [43].

### 2.5. TRAF6

Tumor necrosis factor receptor-associated factor 6 (TRAF6), a ubiquitin ligase in the RING family, is a key bridging molecule of the NF-*κ*B pathway and plays an important role in the regulation of osteoclast formation. Previous studies have shown that TRAF6-deficient mice have bone abnormalities and osteosclerosis [44]. TRAF6 is essential for RANKL signaling and osteoclast differentiation. RANKL recruits TRAF6 binding to E2 ligase Ubc13/Uev1A which promotes site-specific autoubiquitination, thus activating the IKK/NF-*κ*B and JNK/SAPK pathways which promote osteoclast differentiation [45, 46].

### 2.6. TRIM Family

#### 2.6.1. TRIM16

The TRIM protein family includes about 75 proteins with E3 ligase activity and has multiple functions in proliferation, differentiation, apoptosis, carcinogenesis, and autophagy [47]. TRIM16, which belongs to the TRIM family, does not have a RING domain but has E3 ubiquitin ligase activity [48]. A study has shown that TRIM16 and Galectin-3 coregulate the osteogenic differentiation of hBMSCs [49]. Furthermore, TRIM16 reduces CHIP, which reduces CHIP-mediated RUNX2 degradation, thus promoting osteogenic differentiation of hPDLSCs [50].

#### 2.6.2. TRIM21

Tripartite motif containing 21 (TRIM21) is a member of the TRIM protein family with E3 ubiquitin ligase activity. TRIM21 modulated the osteogenic process of MSCs by acting as an E3 ubiquitin ligase to mediate the K48-linked ubiquitination of Akt and cause degradation [51].

#### 2.6.3. TRIM33

Triplex protein 33 (TRIM33) is a member of the TRIM family and a RING type E3 ubiquitin ligase. TRIM33 acts as a positive regulator of osteoblast differentiation in the BMP pathway and its action is mediated by its interaction and activation with Smad1/5 [52]. In addition, TRIM33 protects osteoblasts against oxidative stress-induced apoptosis in osteoporosis by inhibiting ubiquitination and degradation of FOXO3a [53].

#### 2.6.4. TRIM38

Triplex protein 38 (TRIM38) is a member of the TRIM family and a RING type E3 ubiquitin ligase. TRIM38 is involved in various cellular processes such as proliferation, differentiation, apoptosis, and antiviral defense. TRIM38 regulates the NF-*κ*B pathway involved in osteoclast and osteoblast differentiation through ubiquitination and degradation of TGF-Beta Activated Kinase 1 (MAP3K7) Binding Protein 2 (TAB2) protein. Overexpression of TRIM38 in osteoclast precursor cells attenuates RANKL-induced NF-*κ*B activation and osteoblast proliferation and differentiation. Ectopic expression of TRIM38 in osteoblast precursors negatively regulates NF-*κ*B activation and promotes BMP2-induced I*κ*B*α* phosphorylation and degradation for osteoblast differentiation [54].

### 2.7. RNF40

RNF40, a RING family of E3 ubiquitin ligases, monoubiquitinates histone H2A at K119 or H2B at K120, is known to function in transcriptional elongation, DNA double-strand break (DSB) repair processes, maintenance of chromatin differentiation, and exerting tumor suppressor activity [55]. A recent study has found that RNF40-driven H2B monoubiquitination is important for bone integrity in osteoblasts. RNF40 expression is essential for the early stages of lineage specification but is dispensable in mature osteoblasts [56, 57].

### 2.8. RNF146

RNF146 is a RING domain E3 ubiquitin ligase. Mice lacking RNF146 develop a syndrome similar to craniosynostosis dysplasia (CCD) [58]. AXIN is a key node in the Wnt pathway, and RNF146 controls the Wnt/*β*-linked protein pathway through ubiquitination of its substrate AXIN to inhibit osteolysis [59]. 3BP2 is the bridging protein required for the activation of SRC tyrosine kinases and coordinates the attenuation of *β*-linked proteins, which are necessary for osteoclast development. RNF146 also affects bone remodeling via 3BP2 ubiquitination. Furthermore, by regulating the WNT3a-FGF18-TAZ axis, RNF146 can promote osteoblast differentiation and proliferation [60]. Overall, RNF146 regulates the 3BP2/SRC and Wnt/*β*-catenin pathways on bone metabolism by ubiquitination of 3BP2 and AXIN1.

### 2.9. RNF185

RNF185, a RING type E3 ubiquitin ligase, inhibits osteogenic differentiation of mouse cranial-derived MC3T3-E1 cells. The mechanism is the interaction between RNF185 and Dvl2, a key mediator of the Wnt signaling pathway. RNF185 inhibits Wnt signaling and negatively regulates osteogenesis by promoting ubiquitin and degradation of Dvl2 [61].

### 2.10. NEDD4 Family

#### 2.10.1. NEDD4-1

NEDD4/NEDD4-1, an E3 ubiquitin ligase in the NEDD4 family, is essential for osteoblast differentiation and proliferation. Lack of Nedd4 in preosteoblasts results in reduced cell proliferation and altered osteogenic differentiation. Nedd4 promotes the expansion of osteoblast progenitor cell pools which plays an important role in craniofacial development [62]. NEDD4 promotes bone formation primarily by enhancing TGF-*β*1 signaling. NEDD4 promotes osteoblast proliferation by degrading PTEN and TGF-*β*1-activated pSMAD1, upregulating pSMAD2, and promoting TGF-*β*1 gene expression by upregulating PERK1/2 [63, 64].

#### 2.10.2. NEDD4-2

NEDD4-2/NEDD4L is an E3 ubiquitin ligase in the NEDD4 family. NEDD4-2/NEDD4L is similar to SMURF1 and SMURF2. Under SMAD7 participation, NEDD4-2 mediates its degradation by interacting with T*β*R-I. In addition, NEDD4-2 interacts with SMAD2 and induces its ubiquitinization and degradation. In general, NEDD4-2 negatively regulates the TGF-*β* and BMP signaling pathways [65].

### 2.11. WWP Family

#### 2.11.1. WWP1

WWP1 is a member of the SMURF-like C2-WW-HECT (WW is Trp-Trp and HECT is homologous to the E6-accessory protein) type E3 ubiquitin ligases. WWP1 inhibits osteogenesis by promoting SMAD4 in osteoblasts and RUNX2 ubiquitination [35, 66]. In patients with chronic inflammatory diseases, elevated TNF inhibits bone formation through a variety of mechanisms. Junb protein is a key transcription factor that modulates MSCs to differentiate into osteoblasts. Under TNF-mediated mechanisms, WWP1 targets Junb protein proteasome degradation which inhibits bone formation [67]. In addition, WWP1 negatively regulates bone mass by inhibiting MSC migration and osteoblast differentiation. It was also found that WWP1 expression is lower in young MSCs and increases with aging [68].

#### 2.11.2. WWP2

WWP2 is a member of the SMURF-like C2-WW-HECT type E3 ubiquitin ligases, which promotes Sox6 expression through monoubiquitination of Goosecoid under the transcriptional regulation of Sox9 and then promotes craniofacial development [69]. Besides, both WWP2 and Med25 could enhance Sox9 transcriptional activity [70]. Moreover, WWP2 promotes osteogenesis by enhancing RUNX2 through nonproteolytic monoubiquitination [71].

### 2.12. MDM2

MDM2 is an important negative regulator of p53 and an E3 enzyme, which promotes p53 degradation by p53 ubiquitination. P53 is an important tumor suppressor gene in the apoptosis pathway. P53, as a transcription factor, regulates cell cycle arrest, DNA repair, and apoptosis [72]. MDM2 suppresses the action of p53 on the MDM2 gene response element, thus forming a p53-MDM2 regulatory feedback loop. Therefore, in normal cells, p53 is continuously degraded through MDM2-mediated ubiquitination, resulting in a sustained low expression level of p53 [73]. Studies have shown that p53 inhibits osteoblast differentiation and osteoma formation by inhibiting the expression of RUNX2 or Osterix without affecting osteoclast differentiation [74, 75]. MDM2 negatively regulates p53 in favor of RUNX2 activation and is one of the necessary conditions for osteoblast differentiation and appropriate bone formation [76]. Dlx3 is a transcription factor that plays an important role in odontoblast differentiation. MDM2-ubiquitinated Dlx3 upregulates Dspp expression, and MDM2 ubiquitinates P53, which degrades it, reducing the inhibitory effect of mDPCs on odontoblast-like differentiation [77].

### 2.13. SCF^SKP2^

SKP2 is a SCF family protein, and its complex with SKP1 and CUL1 (SCF^SKP2^) is an E3 ubiquitin ligase [78]. This plays an important role in regulating the cell cycle [79]. SKP2 targets RUNX2 for ubiquitin-mediated degradation and thus negatively regulates osteogenesis. Moreover, RUNX2 and SKP2 expression levels in vivo are negatively related [80]. Therefore, SKP2 may be a therapeutic target for osteoporosis.

### 2.14. ITCH

ITCH is a HECT family E3 ligase containing the WW domain. ITCH E3 ubiquitin ligase deficiency in humans and mice leads to syndromic multisystem autoimmune disease [81]. The molecular mechanism of ITCH deficiency leading to autoimmune disease and multiorgan inflammation is related to its negative regulation of JNK and NF-*κ*B signaling pathways [82, 83]. Therefore, the investigators found that Itch negatively regulates osteoblast differentiation from bone marrow mesenchymal stem cells through proteasome degradation of JunB protein [84]. Furthermore, Itch binds to the N-terminal part of NICD through its WW structural domain and inhibits the Notch pathway by promoting Notch ubiquitination through its HECT ubiquitin ligase structural domain [85]. Itch deficiency leads to increased expression of the Notch signal pathway and reduced differentiation of MSCs into osteoblasts, therefore resulting in osteopenic bone phenotype [86]. A study also noted an increase in osteoclasts in the bone marrow of ITCH−/− mice. One of the mechanisms is that ITCH promotes the deubiquitination of TRAF6 by recruiting CYLD to TRAF6 signal transduction complexes. TRAF6 plays an important role in RANKL signal transduction in osteoclasts and osteoclast precursors (OCP). Thus, deubiquitinated TRAF6 negatively regulates osteoclast formation via the RANKL signaling pathway [87].

### 2.15. CHIP

The carboxyl terminus of Hsp70 interacting protein (CHIP or STUB1) is an E3 ligase that regulates the stability of several proteins involved in different cellular functions. Deletion of the CHIP gene leads to a reduced bone mineral phenotype and increased osteoclast formation. CHIP interacts with TRAF6 to promote TRAF6 ubiquitination and proteasomal degradation, thereby inhibiting TRAF6-mediated NF-*κ*B signaling, and plays an important role in osteoclastogenesis and bone reconstruction [88]. In addition to regulating TRAF6, CHIP inhibits TNF*α*-induced NF-*κ*B signaling by promoting the degradation of TRAF2 and TRAF5 [89].

### 2.16. Cbl-b and c-Cbl

The Cbl (Casitas b lineage lymphoma) proteins are an evolutionarily conserved protein family that includes three different gene products (Cbl or c-Cbl; Cbl-b; and Cbl-c, Cbl-3, or Cbl-SL). Cbl-b and c-Cbl proteins are members of the mammalian CBL (Casitas B lineage lymphoma) family and are also Ring E3 ubiquitin ligases which regulate bone metabolism [90]. The effects of Cbl-b and c-Cbl on bone metabolism have been extensively studied, with the literature suggesting that Cbl proteins control osteoblast proliferation, differentiation, and survival through ubiquitination affecting the RTK-PI3K-AKT axis and other c-Cbl target proteins [6, 91, 92]. In addition, Cbl-b and c-Cbl have some less noticeable regulatory effects on bone metabolism. Osterix (also known as Sp7) is an osteogenic-specific cellular regulator which acts downstream of RUNX2 [93]. It was found that Cbl-b/C-cbl reduced the function of Osterix by degrading Osterix with ubiquitin, which inhibited bmp2-mediated osteogenic differentiation [94]. Cbl-b has been shown to be significantly increased in osteoblasts of denervated mice which inhibits IGF-I-regulated osteogenic differentiation by increasing IRS-1 ubiquitination and degradation during denervation [95].

### 2.17. FBL12

FBL12 is an F-box protein induced by TGF-*β*1. p57^KIP2^ is a cyclin-dependent kinase (CDK) inhibitor (CKI) that plays an important role in cell proliferation and differentiation and affects bone development [96]. Under the stimulation of TGF-*β*1, FBL12 and SCF form the SCF FBL12 complex, which directly ubiquitinates p57^KIP2^ and leads to its degradation, thereby inhibiting osteoblast differentiation [97].

### 2.18. LNX2

Notch signaling regulates proliferation, differentiation, and apoptosis in a cell-cell contact-dependent manner. It plays a crucial role in regulating the proliferation and differentiation of osteoblasts and osteoclasts in skeletal development and homeostasis in vivo [98]. LNX2 is a RING-type E3 ubiquitin ligase, which promotes the activation of ERK and AKT induced by M-CSF and the activation of NF-*κ*B and JNK pathways stimulated by RANKL, which in turn promote osteoclast differentiation. Numb protein is an inhibitor of the Notch pathway and LNX2 binding to Numb mediates its ubiquitinated degradation and inhibits Numb-mediated inhibition of osteoblast differentiation by downregulation of the Notch pathway [99].

### 2.19. Parkin

Parkin (Park2) is a RING-between-RING (RBR) E3 ligase [100]. Parkin can be recruited to mitochondria and mediates mitochondrial autophagy, which is related to the pathogenesis of Parkinson's disease [101]. It reduces ROS levels and inhibits apoptosis in osteoarthritic chondrocytes by promoting mitophagy to eliminate damaged/depolarized mitochondria [102] What is more, Parkin promotes osteoblast differentiation of BMSCs by enhancing autophagy and *β*-catenin signaling pathway [103]. NIPA2 is a selective Mg2+ transporter and helps maintain Mg2+ influx. NIPA2 was found to be associated with the development of type 2 diabetic osteoporosis via the mitophagy pathway. The possible mechanism underlying this is that PINK1/Parkin-mediated mitochondrial autophagy in osteoblasts is regulated by NIPA2, which is regulated by the PGC-1*α*/FoxO3a/MMP pathway [104].

### 2.20. Arkadia

Arkadia, a RING-type E3 ubiquitin ligase, is a positive regulator of the TGF-*β* family of SMAD-dependent signaling pathways. Arkadia promotes BMP-induced osteoblast differentiation by downregulating the BMP-specific negative regulators SMAD6, SMAD7, and c-Ski/SnoN to positively regulate BMP signaling [105].

## 3. UPS Inhibitors and Drugs Regulate Skeletal Cell Fate and Pathology

The most commonly used UPS inhibitors in clinical practice are proteasome inhibitors. In 2003, bortezomib (BTZ) became the first proteasome inhibitor approved by the U.S. Food and Drug Administration (FDA). BTZ has been shown to positively affect bone metabolism in MM and promote bone anabolism [106]. It directly inhibits osteoclastogenesis and promotes osteoblastogenesis [107]. Specifically, BTZ can upregulate BMP-2 expression and prevent the proteolytic degradation of the osteoblast transcription factor RUNX2/Cbfa1 to regulate osteoblast differentiation [33, 108]. BTZ inhibits osteoclast differentiation by inhibiting DKK1, RANKL, and NF-*κ*B pathway activity [109, 110]. Experiments have shown that BTZ decreases skeletal complications of MM and prevents mechanical unloading-induced bone loss and ovariectomy-induced osteoporosis in mice [111–113].

In addition to specially developed UPS inhibitors, some commonly used drugs have also been found to be involved in bone metabolism through the UPS system, including thalidomide, lansoprazole, carnosic acid, melatonin, clomipramine, zoledronic acid, and Vitisin A. The immunomodulatory drug (IMiD) thalidomide was originally considered a teratogenic agent but is now used to treat a variety of clinical indications, including MM. It has been found that the direct target of thalidomide is the Cereblon (CRBN), a component of the cullin-4 RING E3 ligase complex. Thalidomide inhibits the ubiquitination of CRBN, leading to increased cullin-4 RING E3 ligase-mediated degradation of target proteins [114]. Recent studies indicate thalidomide has inhibitory effects on glucocorticoid-induced osteoporosis and ovariectomy-induced osteoporosis in mice, but excessive doses of thalidomide can exacerbate osteoporosis [115, 116]. Lansoprazole, which is one of the most commonly prescribed drugs for the treatment of acid-related diseases, induces TRAF6 polyubiquitination, which then activates the noncanonical TAK1–p38 MAPK pathway and facilitates Runx2-mediated osteoblastogenesis [117]. Carnosic acid (CA) is a phenolic acid compound first found in Salvia officinalis L., which possesses antioxidative and antimicrobial properties [118]. CA dually targets SREBP2 and ERR*α*, thus inhibiting the RANKL-induced osteoclast formation and improving OVX-induced bone loss [119]. Melatonin is a signal molecule that modulates the biological circadian rhythms of vertebrates. Melatonin treatment was found to downregulate TNF*α*-induced SMURF1 expression and then decrease SMURF1-mediated ubiquitination and degradation of SMAD1 protein, leading to steady bone morphogenetic protein-SMAD1 signaling activity and restoration of TNF*α*-impaired osteogenesis [120]. Recent studies have shown that clomipramine (CLP) induces bone loss and osteoporosis by acting on Itch to promote osteoclastogenesis. On the contrary, bisphosphonates, such as zoledronic acid (ZA) and prevent bone loss from CLP treatment [121]. One such mechanism is zoledronic acid- (ZA-) induced osteoclast cell ferroptosis by triggering FBXO9-mediated p53 ubiquitination and degradation [122]. A study found that oral administration of a drug containing (+)-Vitisin A significantly improves bone loss in ovariectomized mice. (+)-Vitisin A inhibits RANKL-induced ubiquitination of TRAF6 and formation of the TRAF6-TAK1 complex which inhibits activation of the IKK/NF-*κ*B/c-Fos/NFATc1 signaling pathway to inhibit osteoclast differentiation [123].

There are also a considerable number of E3 ligase drugs in preclinical or clinical trials [124, 125]. The issue is that most of these inhibitors are more effective in cell culture studies and less effective in animal models and clinical trials. Therefore, further research and technological advances will be required in the future [125].

## 4. Noncoding RNAs Regulate Skeletal Cell Fate through the UPS System

Noncoding RNAs (ncRNAs) include intronic RNAs, microRNAs (miRNAs), long noncoding RNAs (lncRNA), circular RNAs (circRNA), and extracellular RNAs [126]. The ability of ncRNAs to control gene expression makes them viable targets for drug development. To date, several ncRNAs were found to act on E3 ubiquitin ligases to regulate bone metabolism. The lncRNA RP11-527N22.2, named osteogenic differentiation inhibitory lncRNA 1 (ODIR1), acts as a key negative regulator during the osteogenic differentiation of hUC-MSCs through the FBXO25/H2BK120ub/H3K4me3/OSX axis [127]. miR-142-5p promoted osteoblast activity and matrix mineralization by targeting the gene encoding WW-domain-containing E3 ubiquitin protein ligase 1 [128]. miR-25 secreted by BMSC-Exo regulates the ubiquitination degradation of Runx2 by SMURF1 to promote fracture healing in mice [129]. Mesenchymal stem cell-derived exosomal miR-19b represses the expression of WWP1 or Smurf2 and elevates KLF5 expression through the Wnt/*β*-catenin signaling pathway, thereby facilitating fracture healing [130]. BMSC-derived exosomal miR-101 augments osteogenic differentiation in MSCs by inhibiting FBXW7 to regulate the HIF1*α*/FOXP3 axis [131]. Silencing DCAF1 by miR-3175 activated Nrf2 signaling to inhibit dexamethasone-induced oxidative injury and apoptosis in human osteoblasts [132]. miR-764-5p positively regulates osteoblast differentiation from osteoblast progenitor cells by inhibiting CHIP protein translation [133]. In addition, biomaterials have also been used as drug delivery platforms to deliver ncRNA. In this research, regenerative siRNA against WW domain-containing E3 ubiquitin protein ligase 1 (Wwp1) complexed with hybrid nanoparticle (NP) were entrapped within poly (ethylene glycol) (PEG)-based hydrogels and implanted at sites of murine middiaphyseal femur fractures. Results showed that fractures treated with siRNA/NP hydrogels exhibited accelerated bone formation and significantly increased biomechanical strength [134].

## 5. Conclusions

Recognition and understanding of the role of the ubiquitin-proteasome system in osteogenic regulation have gained significance in the past decades. Its discovery has helped us understand the nature of biochemical processes behind major developmental and homeostatic events. Numerous ubiquitin enzymes have been discovered so far, with E3 ubiquitin ligases being the most important and diverse. In this review, we discuss and present the role of E3 ubiquitin ligases in bone metabolism, drawing from historical studies on E3 ubiquitin ligases in bone metabolism, as well as recent findings. They regulate bone metabolism through several key factors and pathways that act on osteogenesis and osteoclast.

Designing therapies that target each component of the UPS in order to treat pathology holds great promise for clinical practice. Some proteasome inhibitors are already in clinical use and have been shown to be effective in the treatment of multiple myeloma. Some of these drugs, such as bortezomib, were found to prevent osteoporosis in mice. The main pharmacological effects of some clinical drugs such as thalidomide, lansoprazole, carnosic acid, melatonin, clomipramine, zoledronic acid, and Vitisin A are not related to the UPS system. However, several recent studies have found that these clinical drugs could affect different E3 ubiquitin ligases, which in turn regulate different bone metabolic pathways. Noncoding RNAs, such as miRNA, lncRNA, and siRNA, have also been used to regulate bone metabolism by targeting the UPS system. However, the application of noncoding RNAs is challenged by their poor stability, poor pharmacokinetics, and potential off-target effect. The use of corresponding biomaterials will greatly improve the therapeutic efficacy of noncoding RNA. But the research in this area is relatively basic, and there is still room for further improvement. Moreover, there are a considerable number of E3 ligase drugs in preclinical or clinical trials. Further research and technological advances such as PROTAC (Proteolysis targeting chimeras) may take the research to a new level [135]. With the further discovery of the mechanisms of the E3 ubiquitin ligases related to bone metabolism, more drugs targeting E3 ligases will be designed for the treatment of skeletal disorders.

## Figures and Tables

**Table 1:** E3 ubiquitin ligases and bone metabolism.

Broad group of ligase	Name	Function	References
HECT	SMURF1	Inhibits osteoblast differentiation and mineralization	[15–23, 25, 26, 28–33]
HECT	SMURF2	Inhibits osteoblast differentiation; enhances osteoclast differentiation; inhibits angiogenesis; stimulates endochondral ossification	[34–39]
HECT	Nedd4-1	Enhances osteogenic differentiation	[62–64]
HECT	Nedd4-2	Inhibits osteoblast differentiation and mineralization	[65]
HECT	WWP1	Inhibits osteoblast differentiation and mineralization	[35, 66–68]
HECT	WWP2	Enhances osteogenic differentiation	[69–71]
HECT	Itch	Inhibits or enhances osteogenic differentiation; inhibits osteoclastogenesis	[84–87]
RING	APC/C^CDH1^	Inhibits osteoblast differentiation and mineralization	[42]
RING	APC/C^CDC20^	Enhances osteogenic differentiation	[41]
RING	TRAF4	Enhances osteogenic differentiation	[43]
RING	TRAF6	Enhances osteoclast differentiation	[44–46]
RING	TRIM21	Inhibits osteogenic differentiation	[51]
RING	TRIM33	Protects osteoblasts against oxidative stress-induced apoptosis in osteoporosis	[52, 53]
RING	TRIM38	Enhances osteogenic differentiation; inhibits osteoclastogenesis	[54]
RING	RNF40	Enhances osteogenic differentiation	[56, 57]
RING	RNF146	Enhances osteogenic differentiation; inhibits osteoclastogenesis	[58–60]
RING	RNF185	Inhibits osteoblast differentiation and mineralization	[61]
RING	Mdm2	Enhances osteogenic differentiation	[76, 77]
RING	Cbl-b and c-Cbl	Inhibits osteoblast differentiation and mineralization; enhances osteogenic differentiation	[6, 91–95]
RING	LNX2	Enhances osteoclast differentiation	[99]
RING	Arkadia	Enhances osteogenic differentiation	[105]
B-box	TRIM16	Enhances osteogenic differentiation	[49, 50]
F-box	SCF^Skp2^	Inhibits osteoblast differentiation and mineralization	[80]
F-box	FBL12	Inhibits osteoblast differentiation and mineralization	[96, 97]
U-box	CHIP	Inhibits osteoblast differentiation and mineralization; inhibits osteoclastogenesis	[88, 89]
RBR	Parkin	Enhances osteogenic differentiation	[102, 103]
